# Effectiveness of Continuous Glucose Monitoring on Short-Term, In-Hospital Mortality Among Frail and Critically Ill Patients With COVID-19: Randomized Controlled Trial

**DOI:** 10.2196/67012

**Published:** 2025-02-07

**Authors:** Jiawei Shang, Ziming Yuan, Zuoyan Zhang, Quanhong Zhou, Yan Zou, Wei Wang

**Affiliations:** 1 Department of Intensive Care Medicine Shanghai Sixth People’s Hospital Affiliated to Shanghai Jiao Tong University School of Medicine Shanghai China

**Keywords:** intermittently scanned continuous glucose monitoring, isCGM, COVID-19, in-ICU mortality, continuous glucose monitoring, CGM, point of care testing, POCT, glucose monitoring, in-hospital mortality, mortality, inpatient mortality, critically ill, frail, SARS-CoV-2, intensive care unit, ICU, exploratory, prospective, randomized, open label, parallel, single center, clinical trial

## Abstract

**Background:**

The use of continuous glucose monitoring (CGM) in the hospital setting is growing, with more patients using these devices at home, especially during the COVID-19 pandemic. Frail and critically ill patients with COVID-19 and previously normal glucose tolerance are also associated with variability in their glucose levels during their intensive care unit (ICU) stay. However, very limited evidence supports the use of CGM in ICU settings, especially among frail patients with COVID-19.

**Objective:**

We aimed to investigate the effectiveness of CGM on ICU-related outcomes among frail and critically ill patients with confirmed COVID-19.

**Methods:**

This was an exploratory, prospective, open-label, parallel, single-center, randomized controlled trial. A total of 124 patients was finally analyzed. The primary outcome was 28-day, in-ICU mortality. The secondary outcome included the length of ICU stay as well as the occurrence of hypoglycemia and severe hypoglycemia events.

**Results:**

The mean age was 78.3 (SD 11.5) years. The mean fasting glucose level and hemoglobin A_1c_ level at baseline were 8.12 (SD 1.54) mmol/L and 7.2% (SD 0.8%), respectively. The percentage of participants with diabetes was 30.6% (38/124). The corresponding hazard ratio of the primary outcome in the intermittently scanned CGM (isCGM) group when compared with the point-of-care testing (POCT) group was 0.18 (95% CI 0.04-0.79). The average length of ICU stay was 10.0 (SD 7.57) days in the isCGM group and 14.0 (SD 6.86) days in the POCT group (*P*=.02). At the end of study period, the mean value of fasting glucose in the isCGM group and the POCT group was 6.07 (SD 0.63) mmol/L and 7.76 (SD 0.62) mmol/L, respectively (*P*=.01). A total of 207 hypoglycemia events (<3.9 mmol/L) was detected, with 43 in the isCGM group and 164 in the POCT group (*P*<.001). A total of 81 severe hypoglycemia events (<2.8 mmol/L) was detected, with 16 in the isCGM group and 65 in the POCT group (*P*<.001). The major adverse event in this study was bleeding in the puncture site, with a total of 6 occurrences in the isCGM group. During the follow-up, none of the participants dropped out because of bleeding in the puncture site.

**Conclusions:**

We found a significant clinical benefit from the use of CGM among frail and critically ill patients with COVID-19. These findings support the use of CGM in the ICU and might help with the extension of application in various in-hospital settings.

**Trial Registration:**

Chinese Clinical Trial Registry ChiCTR2200059733; https://www.chictr.org.cn/showproj.html?proj=169257

## Introduction

Continuous glucose monitoring (CGM) is a method of tracking glucose levels throughout the day and night [1]. CGM systems take glucose measurements at regular intervals, 24 hours a day, and translate the readings into dynamic data, generating glucose direction and rate-of-change reports. It is now widely used, medically prescribed, or commercially available for both patients with and without diabetes [2,3]. Several studies have also suggested that CGM has the potential to become the standard of care for some hospitalized patients, overcoming the limitations of current capillary glucose testing [4,5].

The COVID 19 pandemic continues to have a significant impact on the health and wellness of humans despite significant advances in its diagnosis and treatment [6]. CGM has also been used in the management of patients with COVID-19 [7]. In the United States, the Food and Drug Administration (FDA) issued temporary guidance for expanded use of noninvasive remote monitoring devices that reduce contact between clinicians and patients during the COVID-19 pandemic, effectively allowing CGM systems to be used in hospital settings [8].

Several reports indicate that diabetes and hyperglycemia are linked to worse morbidity and mortality in individuals with COVID-19 [9,10]. On the other hand, frail and critically ill patients with COVID-19 and previously normal glucose tolerance are also associated with variability in their glucose levels during their intensive care unit (ICU) stay, which might be caused by the critical illness itself or certain medication therapies such as glucocorticoids and immunosuppressants. Therefore, maintaining optimal glucose levels in hospitalized patients with frailty and critical illness is a key component of care.

However, very limited evidence supports the use of CGM in ICU settings, especially among frail patients with COVID-19. We thus performed an exploratory, prospective, open-label, parallel, single-center, randomized controlled trial (RCT) to investigate the effectiveness of CGM on short-term, in-hospital mortality among frail and critically ill patients with COVID-19.

## Methods

### Study Design

This is an exploratory, prospective, open-label, parallel, single-center RCT. Eligible participants were recruited from Shanghai Sixth People’s Hospital Affiliated to Shanghai Jiao Tong University School of Medicine at Lingang Campus and were randomly divided into the intervention group (wearing a CGM device) and the control group (routine fingertip measurements). The allocation ratio was 1:1. This trial was registered at the Chinese Clinical Trial Registry (ChiCTR2200059733) before the first recruitment of the participants.

### Ethical Considerations

This trial was approved by the Institutional Review Board of Shanghai Sixth People’s Hospital (approval 2022-KY-054). All participants provided written informed consents, and all data collected were only used in this study. All data collected in this study have been anonymized to ensure participant privacy and confidentiality. Personal identifiers have been removed, and unique codes were used to manage data without any direct link to participants’ identities. No compensation was provided to participants in this study. The research was conducted voluntarily, and participants were informed of this during the consent process.

### Recruitment and Screening

The recruitment process involved identifying potential participants from hospital ICU registries. Hospital databases were systematically reviewed to locate patients meeting the initial eligibility criteria, including age, confirmed COVID-19 diagnosis, and indicators of critical illness. Once potential participants were identified, their medical records were thoroughly examined to verify clinical details, including diagnostic results from real-time, reverse transcription polymerase chain reaction (RT-PCR) assays and assessments of respiratory function, oxygen saturation levels, and pulmonary imaging findings. Following this initial review, clinical evaluations were conducted to confirm the presence of the inclusion criteria. In parallel, exclusion criteria were assessed by reviewing the patients’ mental health status and cognitive function to identify any clinically significant psychosis or cognitive impairment that might interfere with their ability to participate in or comply with the study protocol.

### Study Participants

Eligible participants were frail older adults aged 65 years or older who were diagnosed with COVID-19 and critical illness. COVID-19 cases were confirmed by real-time RT-PCR assay of nasal and pharyngeal swab specimens. Critical illness was defined as patients with one of the following conditions: shortness of breath, respiratory rate ≥30 times/min; oxygen saturation ≤93% in a resting state; arterial oxygen partial pressure/oxygen inhalation concentration ≤300 mm Hg; pulmonary imaging showing that the lesions have significantly progressed >50% within 24-28 hours; any occurrence of respiratory failure and requiring mechanical ventilation; or shock, combined with other organ failure. Patients were excluded if they had any clinically significant psychosis or cognitive impairment; they were also excluded if they were unlikely to comply with or complete the study.

### Interventions

The patients in the intervention group were instructed on the use of an intermittently scanned CGM (isCGM) device (Freestyle libre; Abbott Diabetes Care). The sensor was implanted in a site with sufficient subcutaneous fat on the back of the upper arm, and this site can remain flat (without bends or wrinkles) during the participants’ activities. Scars, moles, obesity lines, or lumps were avoided. One sensor lasted no longer than 14 days. If the sensor was expired or malfunctioned, a new one would be immediately implanted until discharge or death. Data were extracted and processed by using a scanner (by swipes) and auxiliary software. The frequency of the swipes followed the routine care during ICU stays at 4 hours intervals or whenever necessary. Data extracted from the CGM device included the mean glucose levels, coefficient of variance (CV), and times of severe hypoglycemia and hypoglycemia (<2.8 and <3.9 mmol/L, respectively) every day. The patients in the control group received a routine fingertip measurement (point-of-care testing [POCT]) for glucose levels. The frequency was consistent with the CGM swipes, ranging from every 1 hour to 4 hours when necessary. If a patient encountered hypoglycemia, more frequent testing of swipes and POCT were done. The mean glucose levels and CV were calculated based on the fingertip glucose profiles.

### Sample Size Calculations

According to a previous case-control study [11], the composite adverse COVID-19 outcome (defined as progression to critical illness or death) occurred in 46% of patients in the isCGM group compared with 66% in the POCT group. Therefore, this study can achieve 80% statistical power at the level of α=.05 by including 50 patients in each group to achieve the superiority end point.

### Randomization, Treatment, and Follow-Up During ICU stay

The participants were randomly assigned to the intervention and control groups in a 1:1 ratio at the time of admission to the ICU. The randomization sequence was generated by the staff. No blinding was applied during the study period. Anthropometric and clinical data were collected from the patients’ self-reporting or the electronic medical records at baseline and at the end of their ICU stay or death after the intervention. These data included sex, height, weight, laboratory measurements, and history of diseases and medications. BMI was calculated as the weight in kilograms divided by the squared height in meters. The treatments of COVID-19 among critically ill patients were prescribed according to the diagnosis and treatment guideline for COVID-19 (ninth version) in China. For all patients received intravenous glucocorticoids, insulin was initiated when the glucose level was persistently higher than 13.9 mmol/L. The average daily dosages of glucocorticoids (converted to the dosages of prednisone) and insulin were also collected.

### Outcomes

The primary outcome was 28-day mortality in the ICU. The secondary outcome included the length of stay in the ICU, as well as the occurrence of hypoglycemia and severe hypoglycemia events (<3.9 and <2.8 mmol/L, respectively).

### Statistical Analysis

The analysis was performed according to the intention-to-treat principle. The data are reported as the mean (SD) unless otherwise stated. One‐way ANOVA was used to analyze differences in population characteristics from baseline to the end of study. A multivariate generalized estimating equation model adjusted for sex and age was used to evaluate influential factors on in-hospital mortality. The Kaplan-Meier survival analysis was used to compare the survival rate between patients in the isCGM and POCT groups. All data were analyzed using R (version 4.0.3; R Foundation for Statistical Computing). A 2‐tailed *P*<.05 was defined as significant.

## Results

### Baseline Characteristics of the Study Participants

**Figure 1:**
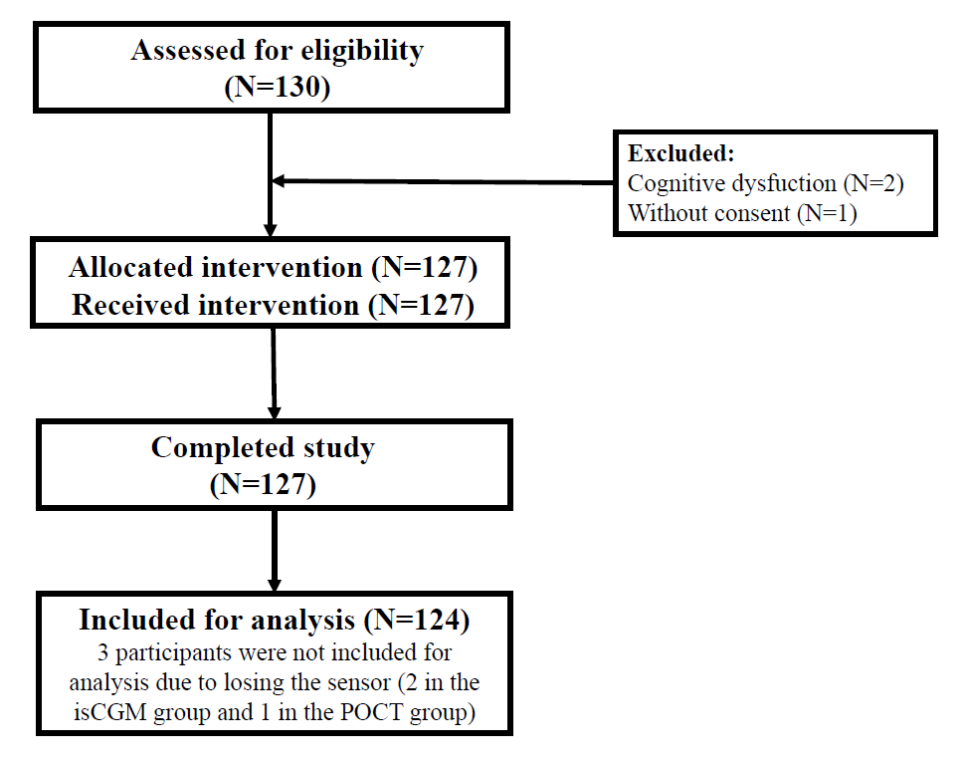
CONSORT (Consolidated Standards of Reporting Trials) flow chart of study. A total of 130 participants were screened for eligibility, and 127 were randomly assigned. All 127 participants completed the study. However, 3 partcipants were not included for analysis due to losing the sensor (2 in the isCGM group and 1 in the POCT group). Thus, the final analysis included 124 participants. isCGM: intermittently scanned continuous glucose monitoring; POCT: point-of-care testing.

Of the 130 participants who underwent screening in this study, 127 (97.6%) were enrolled and randomly assigned. In all, 64 were in the isCGM group while 63 were in the POCT group. Of these 127 participants, a total of 124 (97.6%) completed the study. A total of 2 participants from the isCGM group and 1 participant from the POCT group discontinued the study because they lost the sensor during the study period (Figure 1).

The baseline characteristics are shown in Table 1. The mean age was 78.3 (SD 11.5) years. The mean fasting glucose level at baseline was 8.12 (SD 1.54) mmol/L. The mean hemoglobin A_1c_ level was 7.2% (SD 0.8%). The percentage of participants with diabetes was 30.6% (38/124). The baseline characteristics were well balanced and comparable at baseline.

**Table 1:** Baseline characteristics

Characteristics			isCGM^a^ group (n=62)		POCT^b^ group (n=62)
**Age (years), mean (SD)**			78.8 (11.0)		78.7 (11.9)
**Sex, n (%)**					
	Male	31 (50)		31 (50)	
	Female	31 (50)		31 (50)	
**BMI (kg/m^2^), mean (SD)**			22.2 (3.21)		22.3 (2.93)
**Current smoker, n (%)**			6 (10)		7 (11)
**Heart rate (bpm^c^), mean (SD)**			91.4 (19.0)		86.1 (14.1)
**Respiratory rate (bpm), mean (SD)**			28.0 (3.5)		28.5 (3.7)
**Systolic blood pressure (mm Hg), mean (SD)**			135.5 (25.0)		135.6 (26.3)
**Diastolic blood pressure (mm Hg), mean (SD)**			76.7 (14.5)		75.0 (13.3)
**Comorbidities on admission, n (%)**					
	Diabetes	17 (27)		21 (34)	
	Hypertension	36 (58)		37 (60)	
	Dyslipidemia	4 (6)		5 (8)	
	Coronary heart disease	30 (48)		30 (48)	
	Stroke	35 (56)		38 (61)	
	COPD^d^	35 (56)		34 (55)	
	Chronic kidney disease	16 (26)		21 (34)	
	Cancer	10 (16)		11 (18)	
**Laboratory measurements, mean (SD)**					
	FPG^e^ (mmol/L)	8.10 (1.35)		8.13 (1.67)	
	Hemoglobin A_1c_ (%)	8.3 (1.5)		8.2 (1.4)	
	ALT^f^ (U/L)	63.0 (7.33)		67.4 (6.22)	
	AST^g^ (U/L)	117 (16.0)		203 (14.7)	
	Triglyceride (mmol/L)	0.97 (0.56)		1.01 (0.51)	
	LDL^h^ cholesterol (mmol/L)	3.23 (0.72)		3.19 (0.87)	
	HDL^i^ cholesterol (mmol/L)	0.78 (0.14)		0.82 (0.34)	
	eGFR^j^ (mL/min/1.73m^2^)	60.2 (17.7)		61.0 (18.3)	
	Uric acid (mmol/L)	474.9 (87.3)		480.2 (87.2)	
	C-reactive protein (mmol/L)	62.1 (3.49)		67.6 (5.72)	
	Brain natriuretic peptide (mmol/L)	184.6 (103.7)		150.7 (62.6)	
	Interleukin-6 (pg/mL)	24.3 (4.19)		27.7 (8.37)	
**Use of glucocorticoid, n (%)**			53 (86)		54 (87)
	Daily dose on glucocorticoid^k^, mean (SD)	15.0 (5.23)		15.1 (4.89)	
**Daily dose on insulin, mean (SD)**			15.1 (3.15)		15.4 (2.89)

^a^isCGM: intermittently scanned continuous glucose monitoring.

^b^POCT: point-of-care testing.

^c^bpm: beats per minute.

^d^COPD: chronic obstructive pulmonary disease.

^e^FPG: fasting plasma glucose.

^f^ALT: alanine transaminase.

^g^AST: aspartate aminotransferase.

^h^LDL: low-density lipoprotein.

^i^HDL: high-density lipoprotein.

^j^eGFR: estimated glomerular filtration rate.

^k^Reported as the dosage of prednisone.

### The Changes in Fasting Glucose Levels and Other Glycemic Parameters at the End of the Trial

There was no difference in fasting glucose levels observed between the isCGM and the POCT groups at baseline (mean 8.10 SD 1.35 vs 8.13, SD 1.67; *P*=.43). When compared with the baseline value of fasting glucose, both groups showed significant decreases at the end of the study period (isCGM: mean 8.10, SD 1.35 vs 6.07, SD 0.63; *P*=.001; and POCT: mean 8.13, SD 1.67 vs 7.76, SD 0.62; *P*=.04). At the end of study period, the mean value of fasting glucose in the isCGM group and the POCT group was 6.07 (SD 0.63) mmol/L and 7.76 (SD 0.62) mmol/L, respectively, showing a significant intergroup difference (Table 2; *P*=.01). The daily mean glucose levels (6.93, SD 0.72 vs 8.43, SD 0.52 mmol/L; *P*=.02) and the CV (25.2%, SD 1.23% vs 33.7%, SD 1.15%; *P*=.01) were also found to be significantly lower in the isCGM group than those in the POCT group at the end of the trial. Multivariate generalized linear model defined the reduction of fasting glucose levels as a dependent variable and the control group as the reference. After adjustment of age, sex, and BMI, the results showed that at the end of the study period, the intervention (isCGM) was associated with a larger reduction of fasting glucose levels (–1.69, 95% CI –1.65 to –1.73; *P*<.001).

**Table 2:** Comparisons of glycemic parameters at the end of the trial.

	isCGM^a^ group (n=62), mean (SD)	POCT^b^ group (n=62), mean (SD)	Relative risk or difference (95% CI)	*P* value
Fasting glucose (mmol/L)	6.07 (0.63)	7.76 (0.62)	–1.69 (–1.65 to –1.73)	.01
Daily mean glucose (mmol/L)	6.93 (0.72)	8.43 (0.52)	–1.50 (–1.47 to –1.53)	.02
CV^c^ (%)	25.2 (1.23)	33.7 (1.15)	0.75 (0.71 to 0.79)	.01

^a^isCGM: intermittently scanned continuous glucose monitoring.

^b^POCT: point-of-care testing.

^c^CV: coefficient of variance.

### Mortality in the ICU

**Figure 2:**
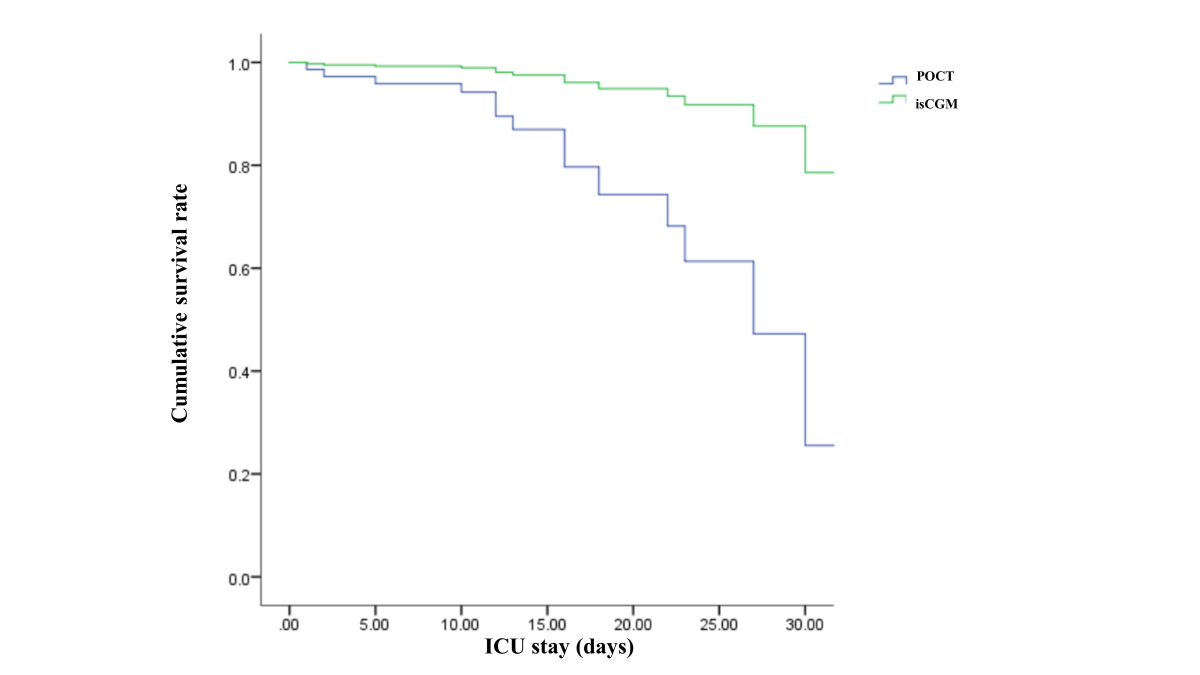
The cumulative survival rate between the POCT and isCGM groups (*P*＜.05). The corresponding hazard ratio of mortality in the ICU in the isCGM group when compared with the POCT group was 0.18 (95% CI 0.04-0.79). ICU: intensive care unit; isCGM: intermittently scanned continuous glucose monitoring; POCT: point-of-care testing.

A total of 15 death events occurred during the study period, with 2 in the isCGM group and 13 in the POCT group. The corresponding hazard ratio of mortality in the ICU in the isCGM group when compared with the POCT group was 0.18 (95% CI 0.04-0.79; Figure 2). The average length of ICU stay was 10.0 (SD 7.57) days in the isCGM group and 14.0 (SD 6.86) days in the POCT group, with a significant *P* value for comparison (*P*=.02).

### Hypoglycemia Events

A total of 207 hypoglycemia events (<3.9 mmol/L) was detected, with 43 in the isCGM group (0.0006 times per patient per day) and 164 in the POCT group (0.002 times per patient per day; *P*<.001). A total of 81 severe hypoglycemia events (<2.8 mmol/L) was detected, with 16 in the isCGM group (0.0002 times per patient per day) and 65 in the POCT group (0.0008 times per patient per day; *P*<.001).

### Adverse Event

The major adverse event in this study was bleeding in the puncture site, with a total of 6 occurrences in the isCGM group. During the follow-up, none of the participants dropped out because of bleeding in the puncture site.

## Discussion

In this exploratory, prospective, open-label, parallel, single-center RCT, we observed a significant improvement on the prognosis of frail and critically ill patients with COVID-19, with fewer in-hospital death events and hypoglycemia events. These findings suggest that the use of isCGM during ICU stay under the COVID-19 pandemic was a protective way to help improve the survival rate among the extremely frail patients.

To date, this might be the first RCT on the use of CGM in the ICU setting among patients with COVID-19, although it was a single-center study with limited sample size. Evidence on the in-hospital use of CGM, especially in the ICU, is still limited. Most studies are observational with a diverse population [12]. While CGM is an efficient tool for patients with diabetes, it has extended its use to other specific populations without diabetes within in-hospital settings such as in the ICU [13], during the perioperative period [14], during pregnancy or labor [15], and among frail populations [16]. The continuous efforts are made by the manufacturers of CGM toward more accuracy, painlessness, and being less burdensome. Early in 2020, the FDA announced the feasibility of using CGM during an in-hospital stay. CGM is essential for the care of glycemic management and can reduce the risk of cross-infections and nosocomial infections among health care professionals.

For the use of CGM in the ICU, several observational studies reported the feasibility and accuracy of different CGM systems. We previously reported the suboptimal overall accuracy of an isCGM system (Freestyle Libre) for critically ill patients. The mean absolute relative difference was reported to be 18% [13]. These data allowed us to further investigate the effectiveness of CGM on ICU-related outcomes among frail patients with COVID-19. We found a significant difference in survival between patients with isCGM and those without isCGM. In another observational study at the Montefiore Medical Center, real-time CGM was used on ICU patients with confirmed COVID-19 infection and glycemic variability [17]. The study demonstrated early feasibility, considerable accuracy, and meaningful reduction in the frequency of POCT. One US study also tested the feasibility among patients receiving operations and those who were admitted to the cardiac ICU, and they provided encouraging results with sustained accuracy of CGM even during exposure to vasopressors [18].

This study aims to fill the gap of evidence on the use of CGM in hospital settings. Less evidence is provided from RCTs of CGM under in-hospital settings. Most of the RCTs focused on the glycemic benefit provided by the use of CGM. Two studies found that the use of the Dexcom senor when compared to either POCT or a blinded CGM device could definitely improve the glycemic control by increasing time in target range and reducing time below or above target ranges [19,20]. In this exploratory, prospective, open-label, parallel, single-center RCT, we first reported the huge benefit of CGM on ICU mortality as well as the length of ICU stay among frail and critically ill patients with COVID-19. The use of CGM also provided a timely chance for the clinicians to observe the hyperglycemia and hypoglycemia events and treat them [20]. There was also a significant difference in the hypoglycemia events between the CGM group and the POCT group. This study supported the hypothesis that CGM might help with the improvement of glycemic control and the outcomes beyond glucose levels. More trials or longitudinal studies with long-term follow-ups are still needed to demonstrate the effect of CGM on disease outcomes other than glycemic control.

The major limitation of this study is the small sample size within a single center. In addition, selection bias might also be introduced because we used a specific population of patients with frailty and critical illnesses, and those patients tended to be older. Whether the findings can be translated to other populations needs further validation in a multicenter design and with a large sample. The strength of this study is that it is the first to demonstrate the association of CGM use and adverse ICU outcomes in frail and critically ill patients with COVID-19. The RCT design also strengthens the level of evidence.

In conclusion, we found a significant clinical benefit from the use of CGM among frail and critically ill patients with COVID-19. These findings support the use of CGM in the ICU and might help with the extension of application in various in-hospital settings.

## Appendix

Multimedia Appendix 1 CONSORT (Consolidated Standards of Reporting Trials) checklist.

## Abbreviations

CGM: continuous glucose monitoring
CV: coefficient of variance
FDA: Food and Drug Administration
ICU: intensive care unit
isCGM: intermittently scanned continuous glucose monitoring
POCT: point-of-care testing
RCT: randomized controlled trial
RT-PCR: reverse transcription polymerase chain reaction

## Data Availability

The original data of this study are not publicly available but are available from the corresponding author upon reasonable request. Access to the data will be granted to researchers who provide a clear and justified purpose for their request, in alignment with ethical guidelines and data protection regulations.
